# Case Report: The SIX Cs model integrating empathy: a structured cognitive framework for psychological first aid during acute threat

**DOI:** 10.3389/fpsyg.2026.1700642

**Published:** 2026-02-18

**Authors:** Moshe Uriel Farchi, Tamar Shlezinger

**Affiliations:** Department of Social Work, Research Center for Innovation in Social Work, Tel-Hai University of Kiryat Shmona in the Galilee, Upper Galilee, Israel

**Keywords:** acute stress reactions, children under terror, empathy, psychological first aid, SIX Cs model

## Abstract

This article presents a theory-driven case study examining the integration of empathy within the SIX Cs Model of psychological first aid (PFA) during a real-time, life-threatening event involving two children trapped in a terrorist attack in Israel. Empathy, encompassing cognitive, emotional, and compassionate components, plays a vital role in psychological support; however, its application during acute stress requires a structured approach that preserves cognitive functioning. The SIX Cs Model targets core features of the acute stress response (ASR), including confusion, perceptual narrowing, helplessness, and impaired executive functioning. Using a descriptive, theory-guided analysis of publicly aired segments from a 12-h phone conversation between a social worker (SW) and the children, the conversation was coded according to the SIX Cs components and types of empathy. The findings show how structured empathic communication can improve prefrontal engagement, reduce loneliness, sustain cooperation, and preserve functional behavior during extreme threat. Real-time behavioral indicators are interpreted through neuropsychological mechanisms associated with prefrontal cortex dominance, amygdala regulation, and dopamine-mediated reinforcement. The case provides rare ecological insight into pediatric PFA during acute danger and illustrates the clinical relevance of integrating empathy within cognitive-based emergency intervention models.

## Introduction

Acute civilian emergencies increasingly require structured psychological first aid (PFA) protocols capable of stabilizing individuals during life-threatening events. While medical first aid is widely standardized, evidence-based guidelines for psychological stabilization remain underdeveloped. The SIX Cs Model addresses this gap through a structured cognitive approach aimed at restoring functioning, reducing helplessness, and counteracting maladaptive responses associated with the acute stress response (ASR).

Empathy is widely regarded as fundamental in trauma care; however, under conditions of immediate danger, it must be delivered in a way that supports, rather than overwhelms, cognitive processes. Unmodulated emotional attunement may intensify distress, whereas cognitive and compassionate empathy help to promote prefrontal activation, clarity, and adaptive behavior. Integrating empathic communication into a coherent cognitive model may consequently enhance the effectiveness of emergency interventions.

The present case study offers a uniquely documented opportunity to examine such integration. On 7 October 2023, two young children maintained a continuous 12-h phone connection with a social worker while hiding from terrorists in their home. Selected segments of this interaction were broadcast on national television, providing exceptional ecological validity and an unusual window into real-time pediatric functioning under extreme threat. This article analyzes these publicly available excerpts, linking the observed intervention strategies to the SIX Cs Model, to different forms of empathy, and to underlying neuropsychological mechanisms.

## The SIX Cs model and the acute stress response

The SIX Cs Model was developed as a structured cognitive framework for PFA, targeting core features of the ASR such as cognitive disorganization, perceptual narrowing, helplessness, and impaired executive functioning. Cognitive communication re-engages prefrontal processes through precise, task-oriented dialog that counters confusion and reduces amygdala-driven perceptual narrowing. Challenge introduces brief, achievable tasks that disrupt freezing, promote goal-directed action, and create immediate experiences of competence. Control restores agency through simple, meaningful choices, enhancing functional capacity and self-efficacy while reducing helplessness. Because choosing between options is a logical process, the control component also increases prefrontal cortex dominance during acute stress. Commitment ensures continuous and reliable presence, reducing loneliness while sustaining the individual’s engagement and cooperation with the helper. Continuity structures the sequence of events, reducing fragmentation and preventing maladaptive memory encoding.

Thus, these components support autonomic regulation and functional restoration during acute stress, with accumulating evidence of reductions in anxiety and improved indices of physiological regulation and resilience following SIX Cs-based interventions in experimental and simulated emergency settings (Farchi et al., 2018; 2024; Thayer et al., 2012).

## Empathy in acute stress interventions: a narrative integration

Empathy plays a central role in helping relationships; however, its meaning and function shift dramatically when the context reflects an immediate danger rather than reflective clinical work. During an acute, ongoing threat, when the individual is immersed in ASR, empathy cannot rely on emotional resonance alone. The neurobiological state of ASR, marked by amygdala hyperactivation, perceptual narrowing, cognitive collapse, and a profound sense of helplessness, establishes conditions where unstructured emotional attunement may intensify distress rather than alleviate it. What is required instead is a form of empathy that is both attuned and strategically delivered, capable of supporting executive functioning and enabling the person to think, decide, and act in the midst of overwhelming fear.

Empathy itself is not a single process but a family of related capacities. Research distinguishes between cognitive empathy (the ability to understand another person’s inner state), emotional empathy (the felt resonance with that state), and compassionate empathy (understanding and concern translate into motivated action) (Decety and Jackson, 2004; Batson, 2009; Eisenberg and Strayer, 1987; Singer and Klimecki, 2014). In therapeutic settings, these dimensions may blend naturally. In acute emergencies, however, each operates differently, and the helper’s capacity to flexibly shift among the forms of empathy carries significant neuropsychological consequences. Cognitive empathy typically activates prefrontal cortical regions, thereby supporting clarity, orientation, and the inhibition of panic-driven impulses. Emotional empathy, when carefully calibrated, conveys recognition of distress without amplification. Compassionate empathy creates a sense of relational safety, which in turn moderates autonomic arousal and reduces the destabilizing effects of loneliness. When these forms of empathy are delivered without structure, they may overwhelm the person. When embedded within a cognitive model such as the SIX Cs, they become mechanisms for restoring function.

The interaction between empathy and neurobiology is particularly striking under conditions of extreme threat. Structured, cognitively oriented empathic communication promotes activation of the prefrontal cortex, the neural system most capable of counteracting fear-driven responses originating in the amygdala (Arnsten, 2009; LeDoux, 2012). The prefrontal cortex enables rule-based thinking, task engagement, prioritization, and behavioral organization, all of which are compromised during ASR. Compassionate presence regulates sympathetic arousal by creating a perception of connection rather than abandonment, a factor associated with polyvagal notions of safety and co-regulation (Porges, 2011). Emotional empathy, when used judiciously, enhances cooperation and supports the internalization of the helper’s guidance. At the same time, the successful completion of small tasks, especially when met with empathic reinforcement, activates dopaminergic pathways that increase motivation and counter freezing behavior (Schultz, 2015). Subsequently, these mechanisms illustrate how empathy, far from being an optional add-on, functions as a neurocognitive tool with measurable effects on functioning during extreme threat.

This layered understanding of empathy becomes more concrete when examining through the SIX Cs Model. The model does not treat empathy as a separate therapeutic element but rather as an active ingredient embedded within each component. Cognitive communication relies on cognitive empathy to gage what the person can process at that moment and to formulate clear, concrete language that organizes perception. Challenge tasks depend on emotional empathy to acknowledge fear while gently redirecting the individual toward achievable action. The component of control, which restores agency by offering simple choices, requires cognitive empathy to judge which options are meaningful and manageable; the very act of choosing increases prefrontal dominance and reduces helplessness, one of the strongest predictors of adverse trauma outcomes. Commitment is the most explicitly relational of the components, drawing on compassionate empathy to reduce loneliness, cultivate trust, and ensure sustained cooperation throughout the unfolding danger. Continuity, which structures the temporal and spatial aspects of the event, again draws on cognitive empathy to anticipate what the person is likely to perceive and how to help them integrate these perceptions coherently rather than fragment into disorganized memory traces (McGaugh, 2015).

The relevance of these mechanisms becomes especially clear when applied to the present case. The SW’s communication with the two children reflected a continuous integration of all three empathic modes. When the SW asked precise, concrete questions about the closet, the phones, and the battery level, she was practicing cognitive empathy, enabling the children to regain orientation despite witnessing the killing of their parents. When she validated their fear while directing Michael to perform small actions, such as silencing the phones or locating a charger, she employed emotional empathy to transform paralyzing terror into manageable task engagement. When she reassured them of her unwavering presence, promising to remain with them until they were rescued, she activated compassionate empathy to counter the profound loneliness of being trapped and bereaved. These empathic messages did not occur instead of intervention; they were the intervention. They restored the ability of the children to think, respond, comply with safety instructions, and perform tasks requiring planning and motor sequencing, an extraordinary outcome given the neurobiological constraints of ASR.

Across the 12 h in which the children remained entrapped, the integration of empathy within the SIX Cs was not only evident but essential. The children’s sustained responsiveness, answering questions, making decisions, retrieving a charger, interpreting sounds, and following instructions, can be understood as a behavioral signature of successful modulation of the ASR. Their brains were repeatedly drawn back into organized cognitive frames by empathically calibrated communication. The SW’s presence became both an emotional anchor and a cognitive scaffold, allowing the children to function far beyond what would typically be expected under such conditions. In this sense, empathy, when structured through the SIX Cs, appears not merely as an ethical necessity but as a functional requirement for preserving life and enabling adaptive action during acute, ongoing threat.

## Case study and analytic approach

This article is a single, theory-driven case study based on a real-time, life-threatening event. Analysis relied exclusively on verbatim excerpts publicly broadcast within the Israeli television program Uvda (Channel 12). These excerpts are drawn from an approximately 12-h phone conversation between two children and SW, a person trained in acute trauma intervention and in the SIX Cs Model. The broadcast segments were selected by the program’editorial team and aired with the consent of the family.

A descriptive, theory-guided case analysis was conducted. Each word in the transcript was examined and coded according to the primary SIX Cs components expressed (cognitive communication, challenge, control, commitment, and continuity) and the predominant type of empathy conveyed (cognitive, emotional, or compassionate). This dual coding enabled an integrated interpretation, linking observed behavior, model components, and hypothesized neuropsychological mechanisms. Table 1 summarizes the conceptual integration of empathy within the SIX Cs, and Table 2 illustrates the application of these components in the concrete interaction between the SW and the children.

**Table 1:** Integration of empathic approaches within the SIX Cs Model.

SIX Cs component 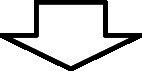	Role of empathy within the component	Neuropsychological mechanisms supported by empathy	Functional outcome during ASR
Cognitive communication	Uses *cognitive empathy* to tailor language to the child’s immediate processing capacity; frames information in short, concrete, and structured units; and avoids emotional overload	Activates prefrontal cortex (PFC), reduces amygdala-driven confusion, supports working memory, and enhances top-down regulation	Restores orientation, improves clarity, and enables the child to follow instructions despite high arousal
Challenge	Uses *emotional empathy* to acknowledge fear while directing the child toward small, achievable tasks that signal competence	Dopamine activation through successful task completion, reduced sympathetic overdrive, and improved motor planning under stress	Increases motivation, counteracts freezing, and restores goal-directed action
Control	Uses *cognitive empathy* to select two simple, meaningful choices; emphasizes agency without overwhelming the child; and reinforces the logical act of choosing	Choice-making strengthens PFC dominance, reduces helplessness, enhances volitional capacity, and supports inhibitory control	Reinstates a sense of agency, counters volitional collapse, and improves decision-making during acute threat
Commitment	Uses *compassionate empathy* to reduce loneliness, affirm connection, and maintain relational engagement; and reinforces the child’s trust in the helper	Reduces cortisol via co-regulation; decreases dorsal vagal shutdown tendencies; stabilizes autonomic function; and promotes a sense of safety	Sustains cooperation; maintains responsiveness over time; and prevents isolation-induced collapse
Continuity	Uses *cognitive empathy* to help the child construct a coherent temporal sequence; supports meaning-making without emotional flooding; and anchors the endpoint of the event	Integrates hippocampal encoding; reduces fragmentation of memory; lowers risk of intrusive recollections; and stabilizes attentional systems	Enhances understanding of the situation; reduces confusion; and supports adaptive processing of the unfolding event

**Table 2:** Transcript of the conversation between the social worker and the children while hiding in the closet.

Speaker	Text	Empathy approach	SIX Cs component/children’s reactions
SW	“*I am with you, and I am not leaving until an Israeli adult comes to get you out of the safe room, okay? Do you have a closet with shelves*?”	Compassionate empathy demonstrating commitment; initial cognitive questions	Commitment; cognitive communication
Michael	“*We have a closet and we are lying on the clothes*”	***	Cooperative response
SW	“*You are amazing, great job, Michael*”	Emotional empathy and positive reinforcement	Challenge; encouraging effective action
Amalia	“*This is Amalia. They’re not here, but they are coming to save us*”	***	***
SW	“*They are coming to save you. Good people are on their way. I am with you until they arrive, okay?*”	Emotional empathy and reassurance; reaffirming commitment	Commitment
SW	“*I want to ask you to put both phones on silent. Can you do that for me, Michael?*”	Cognitive empathy; assigning a simple task	Challenge; promoting cooperation and cognitive engagement
Michael	“*Yes*”	***	Demonstrates cooperation
SW	“*I hear that Amalia is very, very quiet, right? Where are the other siblings?*”	Cognitive empathy; cognitively challenging questions	Cognitive communication
SW	“*Then they tell me they have a sister who is three years old. They tell me she was on dad’s hands, and for them, Abigail is dead too*”	***	Contextual information reported by the SW
Michael	“*We’re not sure she’s dead because she was holding onto my dad, and then she also fell to the floor*”	***	***
Amalia	“My mom and dad are dead because they were shot”	***	***
SW	“*Michael, would you prefer I call in ten minutes, or do you prefer that we stay connected on the line the entire time?*”	Cognitive empathy; offering choice to restore agency	Control
Michael	“*We prefer to stay connected*”	***	Expresses desire for continuous support
SW	“*You prefer to stay connected, so we will stay connected the whole time*”	Compassionate empathy; reinforcing commitment and control	Commitment
SW	“*You do not have to talk all the time. It is okay if I stay with you quietly. For now, just lie quietly on the shelves as you are doing*”	Compassionate empathy; legitimizing silence and maintaining presence	Commitment; continuity
Michael	“*We are… someone outside. Someone is coming to get us, we hear knocking on the door*”	***	Heightened arousal
SW	“*Is someone knocking on the door?*”	Cognitive empathy; clarifying danger	Cognitive Communication
Michael	“*Yes*”	***	***
SW	“*Do not open the door, do not open the door. I am passing on the message. Do not answer whoever is knocking. Lie quietly, okay?*”	Compassionate empathy; safeguarding instructions	Commitment; Control
SW	“*I am with you. You are not alone, okay*?”	Compassionate empathy	Commitment
SW	“*It is good that you are not answering. I know you are with me*”	Compassionate empathy; reinforcing cooperation	Commitment
SW	“*Are there more knocks on the door? Answer with one word, yes or no*”	Cognitive empathy; simplifying communication demands	Cognitive Communication
Michael	“*No*”	***	***
SW	“*It is important for me that you have a phone with battery. Where are your mom and dad and sister? In the living room?*”	Cognitive empathy; clarifying the physical layout	Cognitive Communication; Structuring the event
Michael	“*My mom is in the same room we are, and my dad and Abigail are outside*”	***	***
SW	“*Dad and Abigail are outside, and mom is inside your safe room?*”	Cognitive empathy; structuring the event timeline	Continuity
Michael	“*Yes*”	***	***
SW	“*Okay, so when you come out of the closet, you will see mom?*”	Cognitive empathy; helping create chronological understanding	Continuity
Michael	“*Yes*”	***	***
SW	“*How much battery is left on the phone?*”	Cognitive empathy; assessing resources	Cognitive communication
Michael	“*57*”	***	***
SW	“*Michael, do you know where the phone charger is?*”	Cognitive empathy; prompting action	Challenge
SW	“*Do you feel able to run, even though you will see your mom?*”	Emotional empathy; preparing for exposure while promoting effective action	Challenge
Michael	“*Now Amalia is awake*”	***	***
SW	“*She woke up. Good. Do you want to use this moment to run and get the charger? But make sure you are not seen through the windows*”	Cognitive empathy; encouraging effective action	Challenge
Michael	“*She does not want me to get the charger*”	***	Sibling attachment
SW	“*Amalia is afraid you will go away?*”	Emotional empathy; validating fear	Cognitive communication
Michael	“*Yes*”	***	***
Michael	“*I brought it*”	***	Successful task completion
SW	“*You brought it. Michael, there is no one braver than you*”	Emotional empathy; reinforcing resilience	Challenge; positive reinforcement

SW, Social worker. Full name withheld to preserve anonymity as SW is one of the authors.

## Case description

On 7 October 2023, during a terrorist attack in Israel, 2 children, Michael (8.5 years) and Amalia (6 years), were trapped in a safe room after witnessing the fatal shooting of their parents and the presumed death of their younger sister. While hiding inside a closet within the safe room, they maintained a continuous phone connection for approximately 12 h with the SW. Selected segments of this conversation, totaling several minutes, were later broadcast on national television as part of an investigative feature on the event.

This analysis is based solely on the publicly aired material, supplemented by contextual information available in the public domain. According to these sources, upon rescue, the children were found alert and communicative and were able to follow instructions. They were reunited with their younger sister Abigail, who had been abducted and returned from captivity several months later. The children now reside with extended family members, and the SW continues to maintain supportive contact with them. While no formal clinical assessments of the children’s long-term psychological status are available within the public record, their immediate behavioral functioning at rescue suggests preserved cognitive and behavioral organization despite extreme exposure.

The table shows a systematic and reciprocal link between empathy and the SIX Cs. Cognitive, emotional, and compassionate empathy each support distinct neuropsychological processes that stabilize functioning under acute stress (Shamay-Tsoory, 2010; Singer and Klimecki, 2014; Feldman, 2012). Within the SIX Cs, these empathic processes enable each component to achieve its purpose: cognitive empathy organizes perception, emotional empathy mobilizes action, and compassionate empathy sustains connection. At the same time, the structured nature of the SIX Cs channels empathy into focused and regulating forms, consistent with evidence that guided interpersonal input improves executive functioning under stress (Arnsten, 2009). Together, both the empathy and the SIX Cs mutually reinforce one another, creating a coherent mechanism that supports adaptive behavior during acute threat.

## Case study: saving life through the SIX Cs model combined with empathy

The following case study is taken from the television program *Uvda* (Israeli Channel 12), aired on 24 February 2024. With the family’s permission, the program broadcast short segments from a 12-h phone conversation that took place during the terrorist attack on 7 October 2023. The family also consented to the use of the real names of the children involved. The original conversation was conducted in Hebrew and was broadcast as part of the *Uvda* investigation by journalist Ben Shani (Shani, 2024). This case study includes most of the broadcast material, translated into English, and focuses on the functional use of the SIX Cs Model together with empathic approaches.

During the attack, the SW, who was specialized both in acute trauma and in the SIX Cs Model application, engaged with two children, Michael (9 years) and Amalia (6 years). Their parents had been killed, and they believed their younger sister had also been killed. The continuous phone conversation lasted 12 h, from 06:30 in the morning until 18:30 in the evening, until the children were rescued. Approximately 7 min of the conversation was broadcast at selected intervals, with the family’s permission, showing the real-time application of the SIX Cs Model combined with empathic communication.

## Content warning

The following text may cause emotional discomfort to readers. Nevertheless, it is crucial to examine how the SW consistently integrates the principles of the SIX Cs Model together with nuanced empathic approaches. This integration allows the SW to maintain the children’s cooperation, cognitive engagement, and functional behavior throughout the life-threatening ordeal.

## Post-rescue note

After 12 h, rescue forces reached the children and evacuated them safely. Upon evacuation, both children were medically examined and found to be physically stable. They remained focused, cooperative, and oriented during the extraction, continuing to follow instructions from the rescuers. Only after they reached a safe location and were reunited with their extended family did they begin to show emotional expressions related to the event they had endured. Their younger sister, Abigail, aged four, was later returned from captivity in Gaza after 51 days. Both parents had been killed in the attack. The children now live with their uncles as one family unit, and the SW continues to maintain contact with the family.

## Discussion

The analysis of this case provides a rare, ecologically valid illustration of how a structured cognitive model of PFA can integrate different forms of empathy to stabilize children under extreme, continuous threat. The coded excerpts demonstrate that the SW’s communication was not only supportive but also closely aligned with the components of the SIX Cs Model. Cognitive communication anchored the children in reality, reduced confusion, and guided their attention to do specific tasks. Challenge introduced short, feasible actions that disrupted passivity and enhanced mastery. Control was restored by offering the children meaningful choices, which reintroduced agency and engaged prefrontal processes. Commitment, expressed through sustained, compassionate presence, directly targeted loneliness and preserved the children’s willingness to cooperate. Continuity helped to organize their understanding of what had happened and what they were likely to see upon rescue, reducing the risk of fragmented encoding.

From a neuropsychological perspective, the intervention can be conceptualized as a real-time modulation of ASR. The repeated use of cognitively framed empathy and structured tasks likely increased prefrontal cortex engagement and reduced unchecked amygdala dominance (Arnsten, 2009; LeDoux, 2012). Compassionate presence and continuous connection may have contributed to autonomic regulation in line with polyvagal principles (Porges, 2011), while successful task completion, reinforced by empathic praise, likely activated dopaminergic circuits associated with motivation and approach behavior (Schultz, 2015). The emphasis on chronological and spatial organization of events resonates with evidence on memory consolidation, suggesting that such structuring may reduce the likelihood of later intrusive symptoms (McGaugh, 2015).

Clinically, this case underscores that empathy in acute emergencies cannot be reduced to emotional resonance alone. When delivered through the SIX Cs, empathy becomes a structured, multi-layered scaffold that supports cognition, behavior, and survival. The children’s ability to remain responsive, follow instructions, and perform tasks for many hours in the midst of lethal danger illustrates the potential impact of such an approach.

A central implication of this case is that the children’s preserved functioning can be understood only through the combined operation of the SIX Cs model and empathy-based communication. Cognitive communication and continuity maintained prefrontal engagement, challenge and control supported agency through achievable tasks and simple choices, and commitment reduced loneliness and sustained cooperation. These elements counter core ASR processes such as attentional collapse, loss of control, and disorganized memory encoding. Empathic attunement further enhanced regulation, as interpersonal safety cues are known to reduce amygdala activation and strengthen prefrontal control under acute stress (Arnsten, 2009; Eisenberger and Lieberman, 2004). The resulting 12 h of clarity, cooperation, and behavioral stability are consistent with these mechanisms.

Importantly, beyond its theoretical implications, the effectiveness of the intervention in this case was observed despite the absence of physical proximity, visual cues, and direct non-verbal co-regulation. Under these constrained conditions, empathic communication was delivered primarily through structured verbal interaction. The sustained clarity, cooperation, and functional behavior exhibited by the children throughout 12 h of ongoing threat suggest that when empathy is deliberately organized within a structured framework such as the SIX Cs, it can exert a stabilizing effect even in the absence of typical non-verbal regulatory channels. This observation highlights the robustness of the model and its potential applicability to remote, resource-limited, or high-risk emergency contexts.

## Limitations

Several limitations should be acknowledged. First, the analysis is based solely on publicly broadcast excerpts of a much longer conversation, and important contextual information or additional intervention strategies used by SW may not be fully captured. Second, the case does not include a systematic assessment of physiological indices, vocal tone, prosody, or speech rate, nor does it provide long-term psychological outcome data. Third, this is a single case conducted under highly specific and extreme circumstances and therefore cannot be generalized without caution. Nonetheless, ethical considerations make it impossible to experimentally reproduce such conditions, and the high ecological validity of this case offers insights that are rarely accessible to researchers and clinicians.

## Conclusion

This case study illustrates the clinical and theoretical value of integrating empathy within a structured cognitive model of PFA during acute, life-threatening events. The SIX Cs model, through its components like cognitive communication, challenge, control, commitment, and continuity, targets key impairments of ASR and may be used to restore agency, reduce loneliness, and maintain prefrontal engagement, even in young children under extreme threat. The analysis of real-time behavioral data suggests that such structured empathic interventions may counteract the neuropsychological processes associated with fear, helplessness, and fragmentation. Future work should further examine the implementation of the SIX Cs across diverse populations and contexts, and explore how training in this model could enable both professionals and laypersons to provide effective PFA in emergencies.

## Data Availability

The original contributions presented in the study are included in the article/supplementary material, further inquiries can be directed to the corresponding author.
